# Cerebellar hypoplasia in an Amur leopard cat (*Prionailurus bengalensis euptilura*) with feline panleukopenia virus infection

**DOI:** 10.17221/29/2025-VETMED

**Published:** 2025-12-19

**Authors:** Jang-Hee Han, Jeong-Seop Oh, Seung Yoon Ahn, Jinyoung Kim, Do Na Lee, Young Deok Suh, Dae-Yong Kim, Junghee Yoon, Seong-Chan Yeon

**Affiliations:** ^1^Department of Veterinary Clinical Sciences, College of Veterinary Medicine and Research Institute for Veterinary Science, Seoul National University, Seoul, Republic of Korea; ^2^Department of Veterinary Pathology, College of Veterinary Medicine, Seoul National University, Seoul, Republic of Korea; ^3^Department of Veterinary Medical Imaging, College of Veterinary Medicine, Seoul National University, Seoul, Republic of Korea

**Keywords:** cerebellar malformation, conservation medicine, in utero infection, viral disease, wildlife

## Abstract

Cerebellar hypoplasia caused by feline panleukopenia virus (FPV) is well documented in domestic cats. Still, it remains unreported in wild felid species, including the Amur leopard cat (*Prionailurus bengalensis euptilura*). Understanding the impact of such viral diseases on wild populations is crucial for advancing conservation efforts and protecting wildlife. An orphaned Amur leopard cat exhibiting idiopathic ataxia was rescued. Initial diagnostics, including physical examination, radiography, and blood analysis, yielded no remarkable findings, though its clinical signs indicated an underlying neurological problem. Subsequent real-time polymerase chain reaction tests detected FPV. Magnetic resonance imaging (MRI) revealed brain lesions, including reduced cerebellar parenchyma and cerebrospinal fluid occupying the space where the cerebellum should be located. These findings suggested cerebellar hypoplasia caused by in utero FPV infection. The Amur leopard cat was euthanised owing to its permanent disability, and the necropsy confirmed a markedly shrunken cerebellum. At the same time, histopathology identified decreased cellularity of the molecular and granular layers of the cerebellar cortex. These results coincided with the MRI findings. This report suggests that cerebellar hypoplasia caused by FPV can occur in wild felid species.

Cerebellar hypoplasia (CH) is a common congenital cerebellar malformation in domestic cats, occurring in varying degrees (de Lahunta et al. 2021). The cause of CH in cats is an in utero or early postnatal infection with the feline panleukopenia virus (FPV), also known as feline parvovirus or feline distemper (Schatzberg et al. 2003; Sykes 2014; Pfankuche et al. 2018; de Lahunta et al. 2021). It is a small, non-enveloped DNA virus of the family Parvoviridae that relies on the host’s cell cycle for replication (Garigliany et al. 2016; Pfankuche et al. 2018). It targets developing organs undergoing active cell division, such as the cerebellum during the perinatal period, leading to its shrinkage (Poncelet et al. 2013; Garigliany et al. 2016; Pfankuche et al. 2018). Live FPV vaccination to queens can also introduce developmental issues to the foetal cerebellum (Sharp et al. 1999). As the cerebellum coordinates motor activity by regulating balance, muscle tone, and posture, FPV-induced destruction results in typical ataxia, including a loss of balance, uncoordinated movements, hypermetria, and intention tremors (Schatzberg et al. 2003; Aeffner et al. 2006; March 2006; Poncelet et al. 2013; Sykes 2014; Garigliany et al. 2016; de Lahunta et al. 2021). Although most studies have focused on domestic cats, feline panleukopenia is a highly infectious and deadly viral disease that affects both domestic and wild felids (Kolangath et al. 2023). Accordingly, FPV-induced CH is not restricted to domestic cats, and its potential impact on wild populations should also be considered.

In cats, CH, cerebellar abiotrophy (CA), and Dandy–Walker-like malformation (DWLM) have been documented as developmental cerebellar anomalies (Sharp et al. 1999; Aeffner et al. 2006; Biolatti et al. 2010; Formoso et al. 2023). CH typically presents with clinical symptoms at or shortly after birth, characterised by their non-progressive nature (March 2006; Sykes 2014; de Lahunta et al. 2021). By contrast, CA involves progressive degeneration of cerebellar cortex neurons that worsen over time after a normal birth (Biolatti et al. 2010; Mai 2018; de Lahunta et al. 2021). Despite these differences, observing such patterns in free-ranging animals is challenging, making MRI a beneficial modality for differential diagnosis. In CA, MRI reveals progressive atrophy with cerebrospinal fluid (CSF)-filled spaces between folia. Conversely, CH, present from birth, features an underdeveloped cerebellum with poorly formed folia and lacks CSF-filled interspaces on MRI (MacKillop 2011; Mai 2018). DWLM, a rare congenital anomaly in cats, is characterised by a hypoplastic cerebellar vermis that bridges the bilateral cerebellar hemispheres (Regnier et al. 1993; Formoso et al. 2023). Its clinical signs include severe ataxia and erratic limb movement like other cerebellar problems, while the MRI shows vermis agenesis accompanied by cyst-like dilation of the fourth ventricle (Regnier et al. 1993; Mai 2018; Formoso et al. 2023).

The occurrence of brain deformities such as CH poses profound challenges for wildlife practitioners, as they critically undermine an animal’s survival potential in its natural habitat. When assessing the release potential of rescued wildlife, it is essential to consider not only functional recovery but also ecological adaptability, welfare, and long-term post-release survival (Willette et al. 2023). In individuals with physical handicaps such as limb loss or disability, some may be deemed eligible for release following appropriate rehabilitation and adaptation (Diehl and Stokhaug 2013). However, congenital brain malformations impair key functions such as spatial orientation, motor coordination, and social behaviour, which are fundamental for life in the wild. This makes successful reintroduction into the wild unfeasible despite efforts in training or adaptation (de Lahunta et al. 2021; Kashetsky et al. 2021). Consequently, wildlife with such neurological conditions may appear physically normal but are incapable of living independently, rendering them non-releasable. The conservation risk is further magnified when the underlying cause is an infectious disease, as even a single clinical case may indicate a wider epizootic risk and potential spillover to endangered populations (Daszak et al. 2000). Although limited cases exist, these cases warrant attention from a conservation medicine perspective to understand their causes and implications.

The Amur leopard cat (*Prionailurus bengalensis euptilura*), a subspecies of the leopard cat (*Prionailurus bengalensis*), is the only remaining free-ranging felid native to the Republic of Korea (Jo and Baccus 2016). It holds considerable conservation value and is designated as Class II endangered wildlife in the country due to its regional vulnerability (Jo and Baccus 2016). Genetically, this species is close to the domestic cat (*Felis catus*), demonstrated by a theoretical study on hybridisation (Menotti-Raymond et al. 1999). This genetic proximity not only raises concerns about genetic pollution but also implies susceptibility to disease transmission, especially given the growing presence of stray and feral cats (Kim et al. 2010). Therefore, it is plausible that this species may also be susceptible to infectious diseases commonly observed in domestic cats. Indeed, serological evidence of FPV infection in this species has already been reported, yet its clinical aspects remain poorly understood (Kim et al. 2021). In this context, we report the first case of cerebellar hypoplasia associated with FPV infection in an Amur leopard cat, supported by clinical and post-mortem findings.

## Case presentation

**Figure 1:**
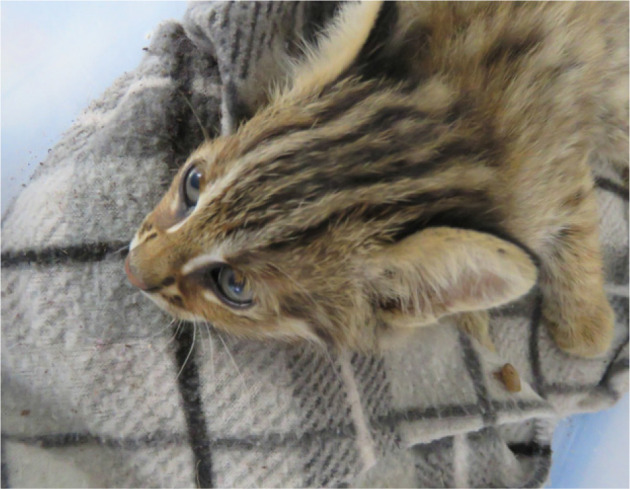
The Amur leopard cat (*Prionailurus bengalensis euptilura*) is designated as class II endangered wildlife in the Republic of Korea This baby Amur leopard cat was rescued with uncoordinated limb movement and hypermetria

An orphaned female Amur leopard cat suspected to be one month old was rescued by a regional wildlife rescue centre (Figure 1). In physical examination, unnatural gait of uncoordinated, poor balanced movement and hypermetria were identified, but there were no visible signs of physical trauma. Radiographic examination was conducted to investigate these symptoms; however, due to the limited resolution of imaging in visualising brain structure, no significant findings were obtained. The patient was rechecked after one week of stabilisation via rehydration, heating, and nutritional support, but their abnormal locomotion has not improved. Blood analysis, including blood smear, complete blood count, and serum biochemistry, did not yield any significant findings related to the symptoms and raised suspicion of a neurological disorder involving the cerebellum.

Although the Amur leopard cat is not a domestic feline, its phylogenetic correlation warranted screening for potential feline-specific pathogens. Initially, a commercial rapid FPV antigen test using its stool was conducted with a kit (Bionote, Gyeong-gi, Republic of Korea), yet it was negative. Due to the possibility of false negatives, fresh stool was collected again and analysed by a commercial laboratory (POBANILAB, Gyeong-gi, Republic of Korea) for more specific diagnostics. Nucleic acids were extracted using the total nucleic acid purification kit (POSTBIO, Guri, Republic of Korea) based on the QIAcube platform (Qiagen). The protocol was customised to achieve optimal conditions. For real-time PCR, 5 μl of nucleic acid was combined with 20 μl of Qiagen master mix for each target, and qPCR or qRT-PCR was performed on an Agilent AriaMx (Agilent, Santa Clara, CA, USA). Target genes for the detection of 19 enteropathogens via real-time PCR included various bacterial, viral, and protozoal pathogens, as well as subtype-specific genes, summarised in Table 1. A C_T_ value of 40 or below was considered positive, and both FPV and *Campylobacter jejuni* were positive.

**Table 1:** The details of real-time PCR for the detection of 19 feline enteropathogens

No	Pathogen	Target gene	Real-time PCR conditions			Results
			PCR protocol	primer/probe concentration	LOD^c^ (based on C_T_ 40)	
1	FPV	*vp2*	PCR^a^	primer: 10 pmole/Rx probe: 5 pmole/Rx	10 copies/Rx	positive
2	FIV	*gag*	PCR	primer: 10 pmole/Rx probe: 5 pmole/Rx	100 copies/Rx	negative
3	FCoV	*M*	RT-PCR^b^	primer: 10 pmole/Rx probe: 5 pmole/Rx	10 copies/Rx	negative
4	Group A rotavirus	*nsp4*	RT-PCR	primer: 10 pmole/Rx probe: 5 pmole/Rx	10 copies/Rx	negative
5	*C. perfringens*	*cpa*	PCR	primer: 10 pmole/Rx probe: 5 pmole/Rx	100 copies/Rx	negative
6	*C. jejuni*	*rimB*	PCR	primer: 10 pmole/Rx probe: 5 pmole/Rx	100 copies/Rx	positive
7	*C. coli*	*gyrB*	PCR	primer: 10 pmole/Rx probe: 5 pmole/Rx	100 copies/Rx	negative
8	*Salmonella* spp.	*invE*	PCR	primer: 10 pmole/Rx probe: 5 pmole/Rx	100 copies/Rx	negative
9	EPEC	*bfpA*	PCR	primer: 10 pmole/Rx probe: 5 pmole/Rx	100 copies/Rx	negative
10	ETEC	*ST/LT*	PCR	primer: 10 pmole/Rx probe: 5 pmole/Rx	100 copies/Rx	negative
11	EHEC	*stx1/stx2*	PCR	primer: 10 pmole/Rx probe: 5 pmole/Rx	10 copies/Rx	negative
12	EIEC	*ipaH*	PCR	primer: 10 pmole/Rx probe: 5 pmole/Rx	100 copies/Rx	negative
13	*C. parvum*	18S rRNA	PCR	primer: 10 pmole/Rx probe: 5 pmole/Rx	100 copies/Rx	negative
14	*G. lamblia*	18S rRNA	PCR	primer: 10 pmole/Rx probe: 5 pmole/Rx	10 copies/Rx	negative
15	*E. histolytica*	18S rRNA	PCR	primer: 10 pmole/Rx probe: 5 pmole/Rx	100 copies/Rx	negative
16	*Cystoisospora* spp.	18S rRNA	PCR	primer: 10 pmole/Rx probe: 5 pmole/Rx	100 copies/Rx	negative
17	*T. foetus*	ITS1	PCR	primer: 10 pmole/Rx probe: 5 pmole/Rx	100 copies/Rx	negative
18	*T. cati*	ITS1	PCR	primer: 10 pmole/Rx probe: 5 pmole/Rx	100 copies/Rx	negative
19	*T. gondii*	RE	PCR	primer: 10 pmole/Rx probe: 5 pmole/Rx	100 copies/Rx	negative

^a^PCR thermal condition: 95 °C, 5 min (95 °C, 10 s – 60 °C, 30 s; 45 cycles); ^b^RT PCR thermal condition: 50 °C, 15 min – 95 °C, 5 min (95 °C, 10 s – 60 °C, 30 s; 45 cycles); ^c^LOD (limit of detection) was determined to be 10-fold serial dilutions of a synthetic plasmid including the target gene of an individual pathogen, based on a C_T_ value of 40

*C. jejuni = Campylobacter jejuni*; *C. parvum = Cryptosporidium parvum*; *C. perfringens = Clostridium perfringens*; *C. coli = Campylobacter coli*; *E. histolytica = Entamoeba histolytica*; EHEC = enterohemorrhagic *Escherichia coli*; EIEC = enteroinvasive *Escherichia coli*; *EPEC* = enteropathogenic *Escherichia coli*; ETEC = enterotoxigenic *Escherichia coli*; FCoV = Feline corona virus; FIV = Feline immunodeficiency virus; FPV = Feline panleukopenia virus; *G. lamblia* = *Giardia lamblia*; *T. cati* = *Toxocara cati*; *T. foetus = Tritrichomonas foetus*; *T. gondii* = *Toxoplasma gondii*

**Figure 2:**
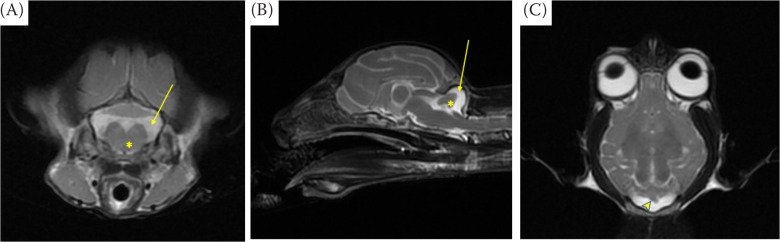
T2-weighted magnetic resonance images from an Amur leopard cat with cerebellar hypoplasia Transverse view of the brain. (B) Sagittal view of the brain. Note the remarkably reduced cerebellum (asterisk) and the accumulation of cerebrospinal fluid (arrow) within the caudal fossa, occupying the space where the cerebellum is typically located. (C) Dorsal view of T2-weighted MRI of the Amur leopard cat. Although the assessment was limited by the overall reduced size of the cerebellum, a structure that appears to be cerebellar vermis (arrowhead) is identified

Magnetic resonance imaging (MRI) was performed under general anaesthesia using a 1.5T MRI scanner (GE Signa 1.5T; GE Healthcare, Illinois, USA) to evaluate the cerebellum. The T2-weighted images (TR/TE 4624/86 on transverse, 3347/112 on sagittal, 3354/115 on dorsal plane) were acquired, revealing a prominently shrunken cerebellum with poor folia formation and the CSF accumulation in the space of the caudal fossa – findings that were not detected on previous radiographic examination (Figure 2A,B). Although the evaluation was limited by the small size, a structure presumed to be the vermis was also identified (Figure 2C). Consequently, the patient was diagnosed with cerebellar hypoplasia. Since the ultimate goal of wildlife rescue is reintroduction into the wild, euthanasia was elected as the most humane option for the Amur leopard cat, given the unfeasibility of release.

**Figure 3:**
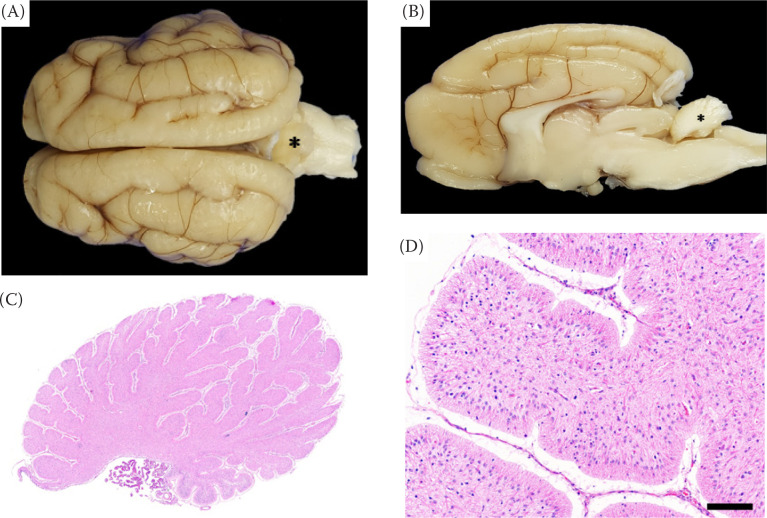
Representative gross and microscopic images of the brain showing cerebellar hypoplasia in the Amur leopard cat (A) Dorsoventral view and (B) midsagittal view of the brain extracted from the calvarium. The cerebellum, indicated with an asterisk, is markedly reduced in size and predominantly composed of white matter (arbor vitae) with minimal grey matter in cross-section. (C,D) Photomicrographs of the cerebellum, stained with H&E. (C) Low magnification showing the cerebellar structure, where the branching pattern of white matter is less distinct due to the diminished molecular and granular layers. (D) High magnification reveals the near absence of molecular and granular layers, with few Purkinje cells and a predominance of the cerebellar medulla Scale bars: (A,B) = 1 cm; (C) = 1 mm; (D) = 100 μm

Following euthanasia, a post-mortem examination was conducted with a particular focus on the central nervous system to investigate the cause of the neurological signs. Gross examination revealed no significant lesions in the thoracic or abdominal organs, including the intestines and lymph nodes, that could be associated with either FPV or *Campylobacter jejuni*. The entire brain, including the cerebellum, was retrieved and fixed in 10% neutral-buffered formalin for seven days. Gross examination of the brain revealed a markedly reduced cerebellum, with a nearly indiscernible cerebellar cortex (Figure 3A,B). Subsequent histopathological examination revealed significantly reduced cellularity in the cerebellar cortical layers, including the molecular and granular layers (Figure 3C,D). As a result, the overall reduced width of the cerebellar cortex caused by the loss of cellularity was considered to be related to cerebellar hypoplasia. When coupled with the clinical signs and concordant MRI findings, these results collectively supported a final diagnosis of CH.

## DISCUSSION

In the present case, sagittal MRI did not show folia with CSF-filled spaces, a cyst-like dilation of the fourth ventricle, while a structure resembling the vermis was visible in the dorsal plane. Based on these MRI findings, CA and DWLM were excluded, and the case was diagnosed as CH. Although CH is an irreversible and untreatable condition, its non-progressive nature allows affected animals to live daily lives as companion pets with appropriate management (March 2006; de Lahunta et al. 2021). However, in this case, the Amur leopard cat is a free-living animal that must preserve its wildness, making successful post-release survival unfeasible. Consequently, euthanasia was adopted based on medical and ethical considerations regarding its future well-being. The necropsy results were consistent with the ante-mortem MRI findings. Histopathological alterations in the cerebellar cortex revealed a loss of cellularity in the molecular and granular layers, which undergo rapid cell division during the in utero or postnatal stage (Poncelet et al. 2013; Garigliany et al. 2016). This suggests that FPV may have targeted and destroyed these cells. Based on this pattern, the CH in this Amur leopard cat closely resembles the FPV-induced pathogenesis in domestic cats (Aeffner et al. 2006). While FPV is known to target rapidly dividing tissues, including the cerebellum, bone marrow, and intestinal crypts, no clinical signs beyond cerebellar abnormalities were observed in this case, and no gross abnormalities were identified during necropsy (Sykes 2014; Garigliany et al. 2016; Pfankuche et al. 2018). This selective vulnerability likely reflects the timing of infection, coinciding with peak cerebellar neuroblast proliferation during the perinatal period (Garigliany et al. 2016). *Campylobacter jejuni*, a Gram-negative bacillus primarily known to affect gastrointestinal tract, was also detected in this case but exhibited no notable clinical signs or lesions upon necropsy. Given that infections in cats are generally self-limited and clinically insignificant, a similar pattern appeared in this patient (Acke 2018).

FPV-induced CH has also been documented in young lions, suggesting that this congenital condition can occur in a range of non-domestic felids (Ma et al. 2022). The Amur leopard cat and lions exhibited pathological findings characterised by reduced cell density in the cerebellar layers and a shrunken cerebellum. In lions, however, more extensive lesions were observed in other brain areas, accompanied by severe neurological symptoms. In contrast, the lesions were confined to the cerebellum in this Amur leopard cat. Hence, FPV may result in varying pathological outcomes across species. Furthermore, the impact of FPV-induced CH may extend beyond felids to other mammalian taxonomic groups. Experimental FPV vaccination resulting in CH in neonatal ferrets indicates that similar effects could appear in mustelids (Duenwald et al. 1971). The detection of FPV in Eurasian badgers further supports its potential link to CH in other mustelid populations (Kim et al. 2021). This implies the need for ongoing FPV surveillance and further research on related neurological disorders in mustelids, primarily to assess the impacts on endangered species and to inform conservation and health management strategies. Moreover, the imprudent vaccination of pregnant wildlife or neonates should be avoided because it may pose a risk of neurological effects on the development of the cerebellum (Sykes 2014).

In this case, the viral infection was confirmed by real-time PCR targeting the *vp2* gene, a highly conserved, host-specific capsid gene widely used in FPV diagnostics (Yang et al. 2022). The *vp2*-based detection thus provides strong molecular evidence of FPV infection, particularly given the gene’s established association with feline-specific strains. Although direct viral detection in brain tissue was precluded by suboptimal preservation, the clinical signs, MRI findings, and histopathological features were highly suggestive of FPV-induced CH (Poncelet et al. 2013). Genomic analysis would have been necessary to elucidate the virus’s phylogenetic background and its connection to wildlife strains; hence, its absence constitutes a notable limitation. Nevertheless, this report constitutes the first clinical documentation of FPV-associated CH in a wild felid in Korea, providing novel insights into the existing body of knowledge. While the findings cannot be generalised from a single case, the integration of molecular diagnostics with clinical and pathological evidence offers a robust foundation for further investigation.

Future studies should pursue direct viral detection in neurologic tissues in conjunction with genetic analyses to clarify pathogenic mechanisms and host-specific responses. Such efforts will be instrumental in informing targeted conservation and infectious disease management strategies for threatened wildlife populations.
